# Editorial: Effects of pesticides on the brain of pollinating insects

**DOI:** 10.3389/finsc.2022.1113610

**Published:** 2022-12-22

**Authors:** Elisa Rigosi, Léa Tison, Albrecht Haase

**Affiliations:** ^1^ Department of Biology, Lund University, Lund, Sweden; ^2^ Département of Plant Health and Environment, National Research Institute for Agriculture, Food and Environment (INRAE), Villenave d’Ornon, France; ^3^ Center for Mind/Brain Sciences, University of Trento, Rovereto, Italy; ^4^ Department of Physics, University of Trento, Trento, Italy

**Keywords:** pesticides, pollinators, neonicotinoids, brain, insects, honey bee, bumblebee

Pollinating insects are crucial to maintaining ecosystem services for both wild and human-selected plants. In agriculture production that is used directly for human food, insect pollination is estimated to be worth 153 billion Euros (1). However, insect pollinators and more generally insects are facing a decline driven by factors such as habitat loss, pesticide use, climate change, land use, invasive species, and agricultural intensification (2–4).

The use of pesticides and the vast ‘accidental’ loss of beneficial insects dates back to the start of modern agriculture (e.g. 5) as non-target insects live and/or visit the same areas as unwanted insect pests. Thus, over their lifespan, non-target insects are exposed (often repeatedly) to intentional or non-intentional cocktails of pesticides *via* multiple routes (air, water, soil, plants, or plant products). Some of these pesticides, for example the majority of insecticides, act as neurotoxins in the brain of insects, they target specific receptors (*e.g.* nicotinic acetylcholine receptors for neonicotinoids) and provoke death *via* spasms and paralysis. However, a more likely scenario for insect pollinators is that they are repeatedly exposed to low-concentration sublethal doses of pesticides. In recent decades, lab, semi-field, and field studies have elucidated the detrimental sublethal effects of pesticides both at individual and colony levels, ranging from disruption of motility, thermoregulation, and sensory perception to more complex functions such as learning and navigation (6–9).

Although many of these substances act directly on the brain of insect pollinators and/or impair their behaviors, very little is known about the neural mechanisms underlying the effects of pesticides. Fundamental questions remain regarding which brain regions are affected most by what compound, which neural and cellular pathways are activated and what the behavioral consequences might be, and which minimal concentrations also cause effects in the long run. To close this knowledge gap, new experimental tools need to be used in the field of ecotoxicology, searching for effects at different scales from the molecular level, looking *e.g.* at gene expression changes, to the cellular level, studying neuronal activity modifications, on the behavioral and cognitive level.

The studies presented in this Research Topic identify and quantify the molecular and cellular pesticide-induced effects underlying previously reported behavioral changes.

Sargent et al. used high-resolution respirometry to investigate how acute exposure to imidacloprid increases the oxygen consumption in the flight muscle of bumblebees and suggest that the change is involved in the reduction of flight activity. They also report an increase in the maximum electron transport capacity in the brain and a trend towards an increased overall oxygen consumption. They suggest follow-up studies on neonicotinoid-induced effects on respiration and energy production.

Christen et al. studied the molecular aspects underlying neonicotinoid-induced effects on foraging and reproduction using radiofrequency tracking technology and gene expression analysis. They found that in bees chronically exposed to neonicotinoids *i.a.* there was a correlation between homing flight duration and the expression of a transcript linked to oxidative phosphorylation. They hypothesize that one reason for prolonged homing flights may be a disruption of energy metabolism.

Using gas-chromatography-coupled electroantennography, Favaro et al. tested *via* the effects of two neonicotinoid pesticides on the ability of honey bees to perceive floral and pheromonal odor compounds. They distinguished between short-term and long-term effects by also testing neonicotinoid-exposed bees the following spring. Treatment with thiacloprid induced changes in antennal responses to specific floral compounds, queen mandibular pheromone, and alarm pheromone components. Treatment effects were generally more prominent in the short term, suggesting that the adverse effects of neonicotinoid exposure may not persist across generations.

Parkinson et al. searched for the mechanisms underlying reported pesticide effects on flying behaviors and showed that chronic exposure to imidacloprid or sulfoxaflor, alone or in a mixture, results in impaired optomotor behavior in the honey bee. This behavioral effect is correlated with increased stress and altered detoxification gene expression in the brain. Sulfoxaflor but not imidacloprid led to a sparse increase in neuronal apoptosis in the optic lobes. They suggest an impairment of the wide-field visual motion neuronal pathway underlying optomotor behaviors.

All presented studies point to a correlation between cellular and sub-cellular mechanisms and impaired behavior. However, as several of the research teams suggest, these findings are by no means final, and often produce new questions. This Research Topic intends to inspire and encourage scientists to follow interdisciplinary approaches when examining the effects of pesticides on the brains of pollinating insects.

## Author contributions

All authors listed have made a substantial, direct, and intellectual contribution to the work and approved it for publication.

## Acknowledgments

AH received financial support by the Autonomous Province of Bolzano (Project CUP B26J16000310003), ER by the Swedish Research Council (VR 2021-03194) and Formas (2018-01218).
